# Peroxisome Proliferator-Activated Receptor-***γ*** in Thyroid Autoimmunity

**DOI:** 10.1155/2015/232818

**Published:** 2015-02-01

**Authors:** Silvia Martina Ferrari, Poupak Fallahi, Roberto Vita, Alessandro Antonelli, Salvatore Benvenga

**Affiliations:** ^1^Department of Clinical and Experimental Medicine, University of Pisa, Via Savi 10, 56126 Pisa, Italy; ^2^Department of Clinical & Experimental Medicine, Endocrinology, University of Messina, Viale Gazzi, Padiglione H, 4 Piano, 98125 Messina, Italy

## Abstract

Peroxisome proliferator-activated receptor- (PPAR-) *γ* expression has been shown in thyroid tissue from patients with thyroiditis or Graves' disease and furthermore in the orbital tissue of patients with Graves' ophthalmopathy (GO), such as in extraocular muscle cells. An increasing body of evidence shows the importance of the (C-X-C motif) receptor 3 (CXCR3) and cognate chemokines (C-X-C motif) ligand (CXCL)9, CXCL10, and CXCL11, in the T helper 1 immune response and in inflammatory diseases such as thyroid autoimmune disorders. PPAR-*γ* agonists show a strong inhibitory effect on the expression and release of CXCR3 chemokines, *in vitro*, in various kinds of cells, such as thyrocytes, and in orbital fibroblasts, preadipocytes, and myoblasts from patients with GO. Recently, it has been demonstrated that rosiglitazone is involved in a higher risk of heart failure, stroke, and all-cause mortality in old patients. On the contrary, pioglitazone has not shown these effects until now; this favors pioglitazone for a possible use in patients with thyroid autoimmunity. However, further studies are ongoing to explore the use of new PPAR-*γ* agonists in the treatment of thyroid autoimmune disorders.

## 1. Introduction

Autoimmune thyroid diseases (AITD) are the most common autoimmune disorders and include Hashimoto's thyroiditis (HT) and Graves' disease (GD), whose clinical features are hypothyroidism and thyrotoxicosis, respectively [1, 2]. The prevalence of AITD is estimated to be about 5% [3, 4]. Several studies have reported an increased incidence of AITD and a progressive decrease in both age at presentation and female to male (F/M) ratio starting in the mid-1990s [5].

A study has evaluated 8397 fine needle aspiration cytologies (FNAC) collected between years 1988 and 2007. The HT increase in frequency started in 1996 (+350% over 1995). Until 1995 there was only one man, but there were 22 men in 2005–2007. These FNAC further support the conclusion that only environmental modifications can explain the marked incidence changes that have occurred in such a relatively short period of time [5].

It has been shown that (1) women have a greater risk than men (5/1, female/male); (2) hypothyroidism from HT is more common with aging; (3) substantial geographic variability in the prevalence of AITD is present; (4) the frequency of antithyroid antibodies is increasing with age; (5) iodine-sufficient areas have higher prevalence of AITD than iodine-deficient ones [6, 7]. AITD are generally of low severity but can affect significantly the quality-of-life (QOL), and they are a cause of considerable medical costs [8].

Cognitive function represents one of the most important parameter of the QOL. The literature available has been reviewed [9]. Conflicting results have been reported on the association between subclinical hypothyroidism and cognitive and health related QOL impairment. Interestingly, it has been frequently reported a reduction in health related QOL in patients with thyroid autoimmune diseases regardless of hypothyroidism or hyperthyroidism [9]. Health-related QOL questionnaires and the disease-specific QOL questionnaire both indicate substantial impairment of QOL in patients with Graves' ophthalmopathy (GO) [10]. GO is a debilitating condition causing facial disfigurement and impaired visual function that have a negative impact on patients' employment, hobbies, and psychosocial function [11].

Epidemiological data suggest that mechanisms that trigger the autoimmune attack to the thyroid are caused by an interaction among environmental triggers and genetic susceptibility leading to the breakdown of immune tolerance and the development of the autoimmune disease [7]. The predominance of AITD in female gender suggests that estrogens are important in the appearance of AITD, such as the immunological changes associated with pregnancy and postpartum. It has been suggested that the presence of cells from one subject in another genetically distinct individual (microchimerism) is one of the endogenous factors linked to AITD [12].

Several environmental risk factors have been identified as follows: radiation, iodine, drugs, smoking, stress, and viruses. These environmental risk factors may activate, in susceptible individuals, the development of AITD [7]. AITD are more prevalent in areas with iodine sufficiency, and in iodine deficient areas supplemented with iodine [13]. Cigarette smoking decreases the risk of overt hypothyroidism but it has been associated with GD and with GO [14, 15].

Thyroid tissue expresses specific selenoproteins; selenium status has an impact on the development of thyroid autoimmunity, and the importance of selenium supplementation in the protection from autoimmune thyroid disorders has been recently emphasized [16].

The contribution of viruses to the occurrence of AITD has been evaluated by many studies with controversial results [17]. An association of HCV infection with AITD has been recently shown both in adults and in children [18, 19]. Moreover, several studies have confirmed a high frequency of autoimmune thyroiditis in patients with mixed cryoglobulinemia and hepatitis C (MC + HCV); in fact, serum antithyroperoxidase (AbTPO), anti-thyroglobulin antibodies (AbTg), and subclinical hypothyroidism were significantly more frequent in MC + HCV patients than in controls. Thyroid disorders observed in HCV infection are characterized by a high frequency of autoimmune thyroiditis and hypothyroidism, in female gender, when high levels of AbTPO are present [7]. More recently, the presence of HCV in the thyroid tissue of HCV patients has been demonstrated, and it has been shown that HCV infects human thyroid cells (ML1), suggesting that HCV infection of thyrocytes plays a role in the association between HCV and AITD [20, 21].

Among drugs, an association of AITD with interferon- (IFN-) *α* therapy in HCV patients has been shown; 40% of HCV patients present thyroid disorders while on IFN-*α* therapy that can manifest as destructive thyroiditis or autoimmune thyroiditis. IFN-*α* induces thyroiditis via both direct toxic effects on the thyroid cells or immune stimulation. HCV and IFN-*α* act in synergism to trigger AITD [22].

Genetic susceptibility to AITD has been shown by (1) the familial clustering of the disease (25% of AITD in siblings of AITD patients); (2) sibling risk ratio of about 17 for AITD; (3) an increased prevalence of thyroid autoantibodies in siblings of AITD patients. Twin studies showed a concordance rate for AITD of 0.5 for monozygotic twins, and the heritability of GD has been calculated to be about 80%, while that of thyroid autoantibodies was about 70% [12]. Many genes have been identified as significantly associated with the AITD and the presence of thyroid antibodies; among these genes whose function is known about 70% are involved in T cells function, suggesting the importance of T lymphocytes in the pathogenesis of AITD [23].

An association between AITD and other autoimmune disorders has been shown. Among organ specific autoimmune disorders, polyglandular autoimmune syndromes are characterized by failure of several endocrine glands (and also nonendocrine organs) induced by an immune destruction of endocrine organs (type 1 diabetes, GD, HT, Addison's disease, vitiligo, alopecia, and hypogonadism were observed in 61%, 33%, 33%, 19%, 20%, 6%, and 5% of these patients, resp.). A common genetic susceptibility is the base of the association of AITD and type 1 diabetes in these patients. These data suggest that patients with AITD should be followed on a regular basis, to evaluate if clinical diseases are present and by serological measurement of organ-specific antibodies [24, 25].

Thyroid autoantibodies and function abnormalities are also present in patients with systemic rheumatologic diseases, such as Sjögren's syndrome (SS), scleroderma, rheumatoid arthritis, systemic lupus erythematosus (SLE), and sarcoidosis [7].

Many studies have shown the presence of a common genetic susceptibility in patients with AITD and systemic autoimmunity; for example, the histocompatibility antigens (HLA) of the haplotypes HLA-B8 and DR3 are associated with both AITD and primary SS in Caucasian patients [26]. Genetic studies in 35 families with cases of SLE concomitant with AITD have identified in 5q14.3-q15 (major* locus* of susceptibility for SLE, also found in AITD) the common link. Also the frequency of HLA-B8 and DR3 is significantly greater in patients with AITD and SLE than in the controls [27, 28].

Also environmental factors could be important in the association of AITD and systemic autoimmune disorders [7].

More recently, several studies have suggested an association of AITD and papillary thyroid cancer (PTC) [29]. In a recent study that analyzed the frequency of PTC, thyroid-stimulating hormone (TSH) levels, and thyroid autoantibodies in 13738 patients, the frequency of PTC was significantly associated with increased levels of TSH [30]. On the contrary, other studies have found that both high TSH and thyroid autoimmunity could represent independent risk factors for malignancy [31]. The increased prevalence of PTC in patients with AITD is clinically important since about 20% of these patients may develop an aggressive disease [32].

The above-mentioned data indicate that patients with AITD should be accurately followed for the appearance of thyroid dysfunctions and thyroid nodules or other systemic or organ autoimmune disturbances during the course of the disease [7].

## 2. Peroxisome Proliferator-Activated Receptor- (PPAR-) *γ*

PPAR-*γ* is a type II nuclear receptor encoded by the* PPARg* gene in humans [33, 34]. Three subtypes of PPARs are known: PPAR-*α*, PPAR-*δ*, and PPAR-*γ* [35]. PPARs form heterodimers with retinoid X receptors (RXRs), thus regulating transcription of various genes. Eicosanoids and free fatty acids are the endogenous ligands of PPARs. PPAR-*γ* acts by (1) controlling glucose metabolism and fatty acid storage, (2) activating genes that promote lipid uptake and adipogenesis by fat cells, and (3) regulating adipocyte differentiation [36]. Of the two known PPAR-*γ* isoforms, PPAR-*γ*1 and PPAR-*γ*2, the first is expressed in almost all tissues except muscle, while the second is expressed particularly in the adipose tissue and in the gut [35]. PPAR-*γ* has been implicated in numerous diseases including obesity, diabetes, atherosclerosis, so that PPAR-*γ* agonists have been used in the treatment of hyperlipidemia and hyperglycemia [37], and cancer. Concerning malignancies, in approximately one-third of follicular thyroid carcinomas the chromosomal translocation t(2;3)(q13;p25) occurs, resulting in the production of the PAX8-PPAR-*γ* fusion protein [38].

There is evidence for an anti-inflammatory role of* PPARg* in inflammatory diseases [39]. In multiple sclerosis (MS), a* PPARg* polymorphism has been shown to be linked to the disease; in fact, the Ala/Ala genotype of the Pro12Ala* PPARg* polymorphism is associated with a delayed onset of disease [40]. In men with coronary artery disease, carriers of the Pro12Ala allele of* PPARg* have less atherosclerosis, vascular morbidity, and mortality [41].* PPARg* also acts as a transrepressor of macrophage inflammatory genes [42].

Animal studies of* PPARg* activating ligands have shown that they have a great anti-inflammatory activity. In a rat model of rheumatoid arthritis, the PPAR-*γ* activating ligands pioglitazone and rosiglitazone (RGZ) were shown to reduce inflammatory bone loss [43]. In a mouse model of SLE, RGZ ameliorated autoantibody production and renal disease [44]. Troglitazone, a PPAR-*γ* agonist, reduced renal scarring and inflammation in a mouse model of renal fibrosis [45]. RGZ decreased expression of the proinflammatory cytokines [interleukin- (IL-) 1b and tumor necrosis factor- (TNF-) *α*] [46], in a rat model of postoperative brain inflammation. The PPAR-*γ* agonists 15-deoxy-D12,14 prostaglandin J2 (15d-PGJ2) and troglitazone both suppress pancreatic inflammation in a rat model of pancreatitis, reducing levels of the inflammatory cytokines IL-6 and transforming-growth-factor-1B [47].

PPAR-*γ* protein has been identified in antigen presenting cells and macrophages. In these cells synthetic PPAR-*γ* agonists (pioglitazone, troglitazone, and RGZ) have been shown to inhibit the secretion of proinflammatory cytokines [48]. The same compounds were demonstrated to decrease the secretion of IL-12, a Th1 inflammatory cytokine, in dendritic cells (that are potent and highly differentiated, professional antigen presenting cells) [49].

Altogether, the above-mentioned studies provide a strong evidence for the anti-inflammatory activity of PPAR-*γ* through its ability to suppress proinflammatory cytokines production in macrophages and dendritic cells [50].

The proven anti-inflammatory action of PPAR-*γ* ligands in animal models of autoimmune diseases has led to the use of PPAR-*γ* agonists in human diseases [51]. In ulcerative colitis, RGZ gave beneficial results in a clinical trial [52]. Other trials on pioglitazone in inflammatory diseases such as rheumatoid arthritis, atherosclerosis, and asthma have been proposed.

Thiazolidinediones [or glitazones, e.g., RGZ, pioglitazone, ciglitazone, etc.] are PPAR-*γ* agonists capable of (1) decreasing insulin resistance; (2) inducing adipocyte differentiation; (3) lowering serum levels of certain cytokines; and (4) inducing antiproliferative mechanisms. Their use in type 2 diabetes has been limited by important cardiovascular side effects, such as edema and heart failure [53–58].

PPAR-*γ* partial agonists activate PPAR-*γ* weaker than thiazolidinediones; they are supposed to have fewer side effects than thiazolidinediones, though conserving their efficacy as hypoglycemic agents. Many of them are natural compounds originating from dietary sources [59, 60].

More recently, PPAR-*γ* has been recognized as playing an important role in the immune response through its ability to inhibit the expression of inflammatory cytokines and to direct the differentiation of immune cells towards anti-inflammatory phenotypes [61–63]. For instance, PPAR-*γ* agonists significantly inhibited the IFN-*γ*-induced expression of the chemokines (C-X-C motif) ligand (CXCL)9, CXCL10, and CXCL11 and inhibited the release of chemotactic activity for the (C-X-C motif) receptor 3 (CXCR3) chemokine receptor-transfected lymphocytes from IFN-*γ*-stimulated endothelial cells (ECs). These data lead to the hypothesis that PPAR-*γ* agonists attenuate the recruitment of activated T cells at sites of Th-mediated inflammation [64].

PPAR-*γ* modulates inflammation through a direct action on the IFN-*γ* inducible chemokines, for example, in the gastrointestinal system [65]. Pioglitazone significantly reduced CXCL10 levels in two models of colitis (dextran sodium sulfate and 2,4,6-dinitrobenzene sulfonic acid-mediated colitis) and dose-dependently reduced CXCL10 levels from activated HT-29 colon epithelial cells and THP-1-derived macrophages [65].

Previous papers have reviewed the evidence of the anti-inflammatory action of PPAR-*γ* agonists in other cells or systems [66–68]. Here, we review the role of PPAR-*γ* in thyroid autoimmunity.

## 3. PPAR-*γ* and Thyroid Autoimmunity

Immunohistochemical expression of PPAR-*γ* was evaluated in histologic sections of thyroid tissue lesions [69], with 6 of 33 samples showing moderate to strong positive staining in focal areas of chronic lymphocytic thyroiditis. The presence of PPAR-*γ* has been also demonstrated in thyroid [70] and orbital tissues of patients with active GO [71]. Indeed, PPAR-*γ* is elevated in the orbital fat of GO patients compared to controls [72, 73]. In another study, the effects of dexamethasone and RGZ on the expression of IFN-*γ* (Th1) and IL-4 (Th2) by activated peripheral CD4(+) and CD8(+) lymphocytes was examined in patients with HT and in healthy control subjects [74]. The inhibition of CD4(+) and CD8(+) IFN-*γ* expression induced by both dexamethasone and RGZ was greater in control subjects than in the HT patients (*P* < 0.05). A more recent study showed that the increased oxidative stress associated with the iodine-induced goiter involution is accompanied by inflammation, and such inflammation can be blocked by 15dPGJ2 through PPAR-*γ*-independent protective effects [75].

**Figure 1:**
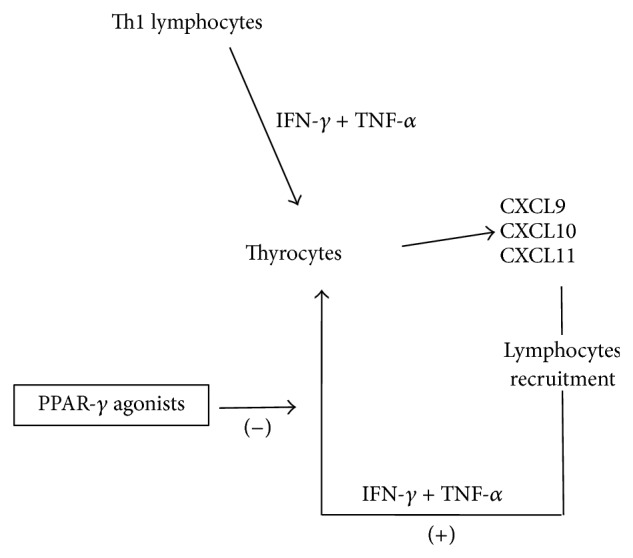
Several cell types (e.g., thyrocytes), under the influence of cytokines (such as IFN-*γ* and TNF-*α*), can modulate the autoimmune response through the production of CXCL9, CXCL10, and CXCL11. These chemokines can induce migration into different tissues of Th1 lymphocytes, which in turn secrete more IFN-*γ* and TNF-*α*, further stimulating the chemokine production by the target cells, thus perpetuating the autoimmune cascade. PPAR-*γ* agonists play an inhibitory role in this process.

In AITD, Th1 immunity and IFN-*γ* play a major role also via the IFN-*γ* inducible chemokines [CXCL11/ITAC, IFN-*γ*-inducible 10-kd protein (IP-10/CXCL10), and monokine induced by IFN-*γ* (MIG/CXCL9)] [76]. These cytokines bind to the CXCR3 chemokine receptor [76]. CXCL10 regulates inflammation by generating directional migration of multiple immune cell types (activated T cells, monocytes, and natural killer cells) [76] and by inducing other cytokines, such as IL-8 and CXCL5 [76]. CXCL10 production is induced by IFN-*γ* in different cells (T lymphocytes, monocytes, fibroblasts, thyrocytes, preadipocytes, and others). In turn, recruited Th1 lymphocytes enhance IFN-*γ* and TNF-*α* release, which stimulate the production of CXCL10, hence creating an amplification feedback loop [76] (Figure 1).

CXCL10 secretion increases with aging [77], and the presence of elevated CXCL10 levels in peripheral liquids is a marker of a Th1-orientated immune response. Furthermore, serum levels and/or tissue expression of CXCL10 is increased in organ-specific autoimmune diseases, such as type 1 diabetes mellitus [78, 79], rheumatoid arthritis [80], SLE [81], systemic sclerosis [82, 83], psoriasis or psoriatic arthritis [84], sarcoidosis [85], HCV-related cryoglobulinemia [79, 86, 87], and other HCV immune-mediated disorders [88–91] and also in cancers [92].

IFN-*γ* dependent chemokines (CXCL9, CXCL10, and CXCL11) are involved also in thyroid disorders, such as HT [93]. CXCL10 serum levels are elevated during the active phase of GD but normalize upon treatment, once euthyroidism has been restored. Similarly, high levels of CXCL9 and CXCL10 are associated with the active inflammation in GO but they diminish after treatment with corticosteroids [94, 95].

Primary cell cultures of thyrocytes, retrobulbar fibroblasts, and preadipocytes from GO patients did not release CXCL9, CXCL10, and CXCL11 at baseline [96, 97], but their secretion was dose-dependently induced by IFN-*γ* alone or combined with TNF-*α*. In turn, the IFN-*γ* + TNF-*α*-stimulated secretion of those chemokines was dose-dependently inhibited by RGZ (0.1–10 M). These data suggest that PPAR-*γ* agonists exert an inhibitory effect in the modulation of CXCR3 chemokines [96, 97] (Figure 1).

Moreover, the cotreatment with IFN-*γ* + TNF-*α* enhanced both the DNA binding activity of the nuclear factor kappa-light-chain-enhancer of activated B cells (NF-kB) in GD thyrocytes and the secretion of CXCL10 [98]. Pioglitazone inhibited dose-dependently the IFN-*γ* + TNF-*α*-induced CXCL10 secretion in thyrocytes, orbital fibroblasts, and preadipocytes from GO patients, while RGZ and pioglitazone reduced the IFN-*γ* + TNF-*α* activation of NF-kB in GD thyrocytes [98].

*In vitro* studies have demonstrated the synergistic effect of either IFN-*α* or IFN-*β* with TNF-*α* on CXCL9, CXCL10, or CXCL11 secretion [99]. PPAR-*γ* agonists were able to modulate the secretion of the IFN-*α* and IFN-*β* stimulated CXCR3 chemokines [99]. In fact, RGZ dose-dependently inhibited the IFNs-stimulated CXCL9, CXCL10, and CXCL11 secretion in thyrocytes (Figure 1).

More recently, our group showed the effects of the IFN-*γ* + TNF-*α*-stimulation and of increasing concentrations of the PPAR-*γ* agonists (pioglitazone or RGZ) on the Th1-chemokine CXCL10 and the Th2-chemokine (C-C motif) ligand (CCL)2 secretion in primary cultures of extraocular muscle (EOM) cells from GO patients [100]. In primary EOM cultures CXCL10 was undetectable in the supernatant; IFN-*γ*, but not TNF-*α*, dose-dependently induced it. In contrast, TNF-*α*, but not IFN-*γ*, dose-dependently induced CCL2. As expected, IFN*γ* + TNF*α* synergistically induced the CXCL10 and CCL2 secretion. However, PPAR-*γ* agonists inhibited the CXCL10 secretion, but stimulated CCL2 secretion. These results suggest that EOM cells play a major role in the inflammation associated with GO, by releasing both Th1 (CXCL10) and Th2 (CCL2) chemokines upon stimulation (Figure 1) [100].

Treatment with pioglitazone was reported to expand the orbital fat in diabetic patients with [101] or without thyroid eye disease [102]. GO patients who carry the Pro(12)Ala* PPARg* polymorphism develop a less-severe and less-active disease [103, 104]. Hence, this polymorphism was proposed to protect from GO development and from a severe course of GO [103, 104].

## 4. Conclusion

PPAR-*γ* is strongly expressed in thyroid tissue of patients with AITD, HT, and GD but also in the orbital tissue (particularly the EOM cells) of patients with GO. In addition, there are enough experimental studies to support the importance of the CXCR3 receptor and cognate chemokines (CXCL9, CXCL10, and CXCL11) in the Th1 immune response and in inflammatory diseases such as AITD.* In vitro* studies have shown that PPAR-*γ* agonists strongly inhibit the expression and release of CXCR3 chemokines in a number of cells, such as thyrocytes, orbital fibroblasts, preadipocytes, and myoblasts.

While RGZ has been withdrawn from the European market by the European Medicines Agency in September 2010, because of the increased risk of heart failure, stroke, and all-cause mortality in old patients [105], so far pioglitazone has not shown these side effects. Pioglitazone, which is commonly used in the treatment of type 2 diabetes [106, 107], has been recently proposed for the treatment of immune-related disorders.* In vivo* studies addressing the use of PPAR-*γ* agonists in AITD patients are ongoing.
